# Systemic treatment of breast cancer with leptomeningeal metastases using bevacizumab, etoposide and cisplatin (BEEP regimen) significantly improves overall survival

**DOI:** 10.1007/s11060-020-03510-y

**Published:** 2020-04-29

**Authors:** Tom Wei-Wu Chen, I-Shiow Jan, Dwang-Ying Chang, Ching-Hung Lin, I-Chun Chen, Ho-Min Chen, Ann-Lii Cheng, Yen-Shen Lu

**Affiliations:** 1 Department of Oncology, National Taiwan University Hospital, 7 Chung-Shan South Rd, Taipei, 10002 Taiwan; 2 National Taiwan University Cancer Center, Taipei, Taiwan; 3 Graduate Institute of Oncology, National Taiwan University College of Medicine, Taipei, Taiwan; 4 Department of Laboratory Medicine, National Taiwan University Hospital, Taipei, Taiwan; 5 Health Data Research Center, National Taiwan University, Taipei, Taiwan

**Keywords:** Bevacizumab, Breast cancer, Chemotherapy, Intrathecal therapy, Leptomeningeal metastasis, Trastuzumab

## Abstract

**Introduction:**

Metastatic breast cancer (MBC) with leptomeningeal metastases (LM) has dismal survival. We aim to determine if modern systemic therapy, especially the bevacizumab, cisplatin, and etoposide (BEEP) regimen, is beneficial to MBC LM patients.

**Methods:**

We excerpted data from a prospectively collected cytopathology database for MBC patients who were diagnosed with LM by positive cerebrospinal fluid cytology. The primary outcome was OS from cytologically confirmed LM until death. Univariate and multivariate analyses were performed to elucidate prognostic factors.

**Results:**

We identified 34 patients with cytologically confirmed LM. Treatments after LM diagnosis included: intrathecal methotrexate (82.4%), systemic chemotherapy (68%; BEEP n = 19, others n = 4), and whole brain radiotherapy (n = 5, 14.7%). Three of seven HER2-positive patients (43%) also received intrathecal trastuzumab. OS was improved in 2014–2016 compared with 2011–2013 (13.57 vs 3.20 months, p = 0.004), when 12/17 (71%) versus 7/17 (41%) patients received BEEP, respectively. In the multivariate model including all treatments, BEEP (HR 0.24, p = 0.003) and intrathecal trastuzumab (HR 0.22, p = 0.035), but not intrathecal methotrexate (HR 0.86, p = 0.78), remained significant prognostic factors.

**Conclusions:**

MBC with LM is treatable—systemic BEEP are efficacious and may improve survival.

**Electronic supplementary material:**

The online version of this article (10.1007/s11060-020-03510-y) contains supplementary material, which is available to authorized users.

## Introduction

Leptomeningeal metastasis (LM) is common in lung, breast, and renal cell cancers and melanomas, and is one of the most devastating metastatic disease scenarios [1]. The incidence of LM in metastatic breast cancer ranges widely, from 5 to 40%, and the prognosis is very poor, with median overall survival (OS) of around 3–4 months from diagnosis [2, 3]. Furthermore, there is evidence of increasing incidence rates of central nervous system (CNS) metastases, including brain parenchyma and possibly leptomeninges, in metastatic breast cancer [4, 5]. Thus, patients with breast cancer LM desperately need new treatments or drug regimens that can improve their prognosis.

Although many potential treatments for LM have been evaluated, results hitherto have been unsatisfactory. Intrathecal chemotherapy, which has the advantage of not needing to penetrate the blood–brain-barrier to reach the cerebrospinal fluid (CSF), has been the favored treatment, but its efficacy remains uncertain [6]; despite the advantages of intrathecal chemotherapy, multiple studies suggested that its efficacy is modest at most [1, 6–8]. Systematic reviews have even suggested that intrathecal methotrexate is associated with increased toxicity, without improving outcomes [6, 9]. Another promising intrathecal treatment for HER2-positive breast cancer with LM is trastuzumab; case reports have shown that intrathecal trastuzumab is a feasible treatment with limited toxicity [10], but more data are needed to determine its actual efficacy.

Although retrospective studies suggest that systemic treatment may improve the survival of breast cancer with LM [9, 11], many prospective trials of systemic treatments, such as temozolomide, have not demonstrated convincing clinical efficacy [12]. We recently devised a combination of the anti-vascular endothelial growth factor monoclonal antibody bevacizumab, plus etoposide and cisplatin (BEEP regimen), which has shown significant activity in patients with breast cancer and brain metastases that progressed after whole brain radiotherapy (WBRT) [13]. We reported promising results of patients with breast cancer LM who responded to the BEEP regimen, supporting the rationale for using BEEP to treat breast cancer patients with CNS metastases [14, 15].

The National Taiwan University Hospital (NTUH) criterion for definitive diagnosis of LM, is positive CSF cytology; this policy has dual benefits: first, it excludes patients with false-positive diagnoses based solely on CNS imaging; second, CSF cytology results can be used to accrue an unbiased dataset of patients with LM in real-world practice. The aim of this study was to assess the effect of systemic BEEP on OS of breast cancer patients with cytologically confirmed LM.

## Methods

We used the NTUH cytopathology database to identify patients from 2011 to 2016 with confirmed breast cancer and CSF cytology results positive for LM. Clinicopathologic data and treatment modalities were excerpted from electronic medical records. The NTUH Research Ethics Committee approved the study under a blanket protocol (201003025R) for analysis of medical data from patients with stage IV breast cancer. Informed consent from the patients was waived for retrospective medical record review studies per the protocol of the NTUH Research Ethics Committee. Estrogen receptor (ER) and human epidermal growth factor receptor-2 (HER2) status were determined based on primary breast tumor samples; patients with ≥ 1% of nuclear immunohistochemistry staining were considered ER positive and HER2 status was defined according to current American Society of Clinical Oncology criteria [16].

The first date of confirmed CSF cytology for malignant cells was considered the index date for treatment and survival. The first intrathecal and systemic treatments received after LM was confirmed were noted. WBRT was defined as having received WBRT within ≤ 30 days of the index date. The BEEP regimen entailed a 21-day cycle of bevacizumab (15 mg/kg) on day 1, followed by cisplatin and etoposide (both 70 mg/m^2^) on day 2, then etoposide (70 mg/m^2^) only on days 3 and 4 [13].

The primary endpoint was OS, defined as the period elapsed from the index date until death or last follow-up, in August 2018. The progression of LM was defined by the deteriorating of neurological symptoms. The time to response was calculated from the index date to the first negative cytology report date; patients with two consecutive negative CSF cytology assessments were considered responsive to LM treatment.

### Statistical analysis

All statistical analyses were performed using SAS, version 9.4 (SAS Institute Inc., Cary, NC, USA). Kaplan–Meier survival curves with 95% pointwise confidence limits and log-rank tests were used to elucidate associations between prognostic factors and OS. Univariate and multivariate Cox proportional hazard models were used to estimate hazard ratios (HRs) and 95% confidence intervals (CIs). To determine which treatments may be most beneficial for patients with LM, all that were significantly associated with OS in univariate analyses, were tested in the multivariate model. A p-value of < 0.05 was considered significant and no multiple comparison corrections were performed.

## Results

### Patient characteristics

From 2011 to 2016, the NTUH cytopathology database recorded 34 cases of breast cancer with cytologically confirmed LM; Table 1 summarizes the patients’ clinicopathologic characteristics. All were females, with median age of 57 (30–80) years at LM diagnosis, and > 80% were initially diagnosed with stage III or IV breast cancer. The median interval from first breast cancer diagnosis to LM was ~ 34.2 months (0.7–149.8) and from metastatic disease diagnosis to confirmed LM was ~ 16.7 months (0–98.9).

**Table 1:** Characteristics of 34 patients with leptomeningeal metastases from breast cancer

Data show median [range] or number (%)	
Year of leptomeningeal metastases diagnosis	
2011–2013	17 (50.0)
2014–2016	17 (50.0)
Age at leptomeningeal metastasis diagnosis	57.0 [30.0, 80.0]
Age at first breast cancer diagnosis	53.0 [26.0, 77.0]
Breast cancer stage at first diagnosis	
II	6 (17.6)
III	15 (44.1)
IV	13 (38.2)
Interval breast cancer diagnosis to leptomeningeal metastases (months)	34.2 [0.7, 149.8]
Interval from stage IV diagnosis to leptomeningeal metastases (months)	16.7 [0.0, 98.9]
Histology	
Carcinoma	4 (11.8)
Ductal	22 (64.7)
Lobular	8 (23.5)
Subtype	
ER+	21 (61.8)
HER2+	7 (20.6)
TNBC	10 (29.4)
Brain metastasis	
Synchronous	12 (35.3)
Metachronous	10 (29.4)
Not present	12 (35.3)
Extra-CNS metastasis	
Any	26 (76.5)
Bone	20 (58.8)
Liver	12 (35.3)
Lung	8 (23.5)
Soft tissue/lymph nodes	2 (5.9)
Stereotactic radiosurgery before leptomeningeal metastases diagnosis	7 (20.6)
Interval from stereotactic radiosurgery to leptomeningeal metastases (months)	3.2 [0.86, 18.6]
Whole brain radiotherapy before leptomeningeal metastases diagnosis	8 (23.5)
Interval from whole brain radiotherapy to leptomeningeal metastases (months)	5.5 [2.58, 24.56]
Treatment after leptomeningeal metastases diagnosis	
Intrathecal methotrexate	28 (82.4)
Systemic therapy	23 (67.6)
BEEP regimen	19 (55.9)
Other regimens^a^	4 (11.7)
Anti-HER2 therapy (% of HER2 + patients)	3 (42.9)
Whole brain radiation therapy (± 30 days)	5 (14.7)

*ER* estrogen receptor, *HER2* human epidermal growth factor receptor-2, *TNBC* triple negative breast cancer, *CNS* central nervous system, *BEEP* bevacizumab, etoposide, and cisplatin

^a^Capecitabine (1), etoposide & cisplatin (1), paclitaxel & gemcitabine (1), bevacizumab, docetaxel & cisplatin (1)

Primary breast tumors were predominantly ER-positive (21/34), HER2-negative (27/34), with 10/34 triple negative. Eight patients (23.5%) had lobular histology. Despite LM, 12/34 patients did not have synchronous parenchymal brain metastases; common metastatic sites besides CNS included bone, liver, lung, and soft tissue/lymph node.

Non-surgical treatments of metastatic brain tumors before LM diagnosis included stereotactic radiosurgery (SBRT) and WBRT. The median interval from previous SBRT and WBRT to LM diagnosis was 3.2 and 5.5 months, respectively.

### Treatment for LM

Patients with LM generally received concomitant multimodal treatments after the index diagnosis; most received at least one dose of intrathecal methotrexate and systemic chemotherapy. The BEEP regimen was the first-line systemic treatment for 19/23 patients, and only 2/23 who received systemic treatment did not receive intrathecal methotrexate. Three of seven patients with HER2-positive breast cancer with LM had also received concomitant intrathecal trastuzumab during the treatment course, but none before the LM index date.

### Survival analysis

**Fig. 1:**
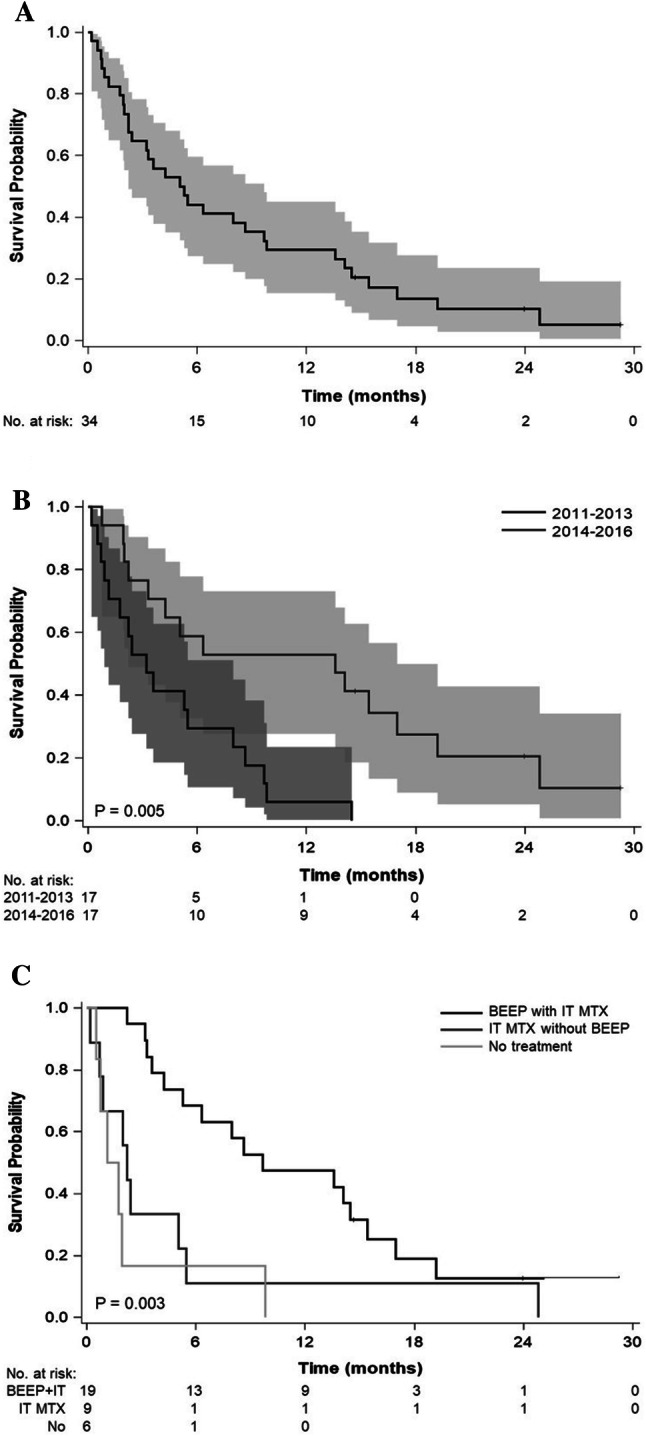
**a** Kaplan–Meier survival curves with 95% confidence intervals for the entire cohort (n = 34). **b** Kaplan–Meier survival curves of patients treated from 2011–2013 vs 2014–2016. **c** Kaplan–Meier survival curves of patients who received different treatments. *BEEP* bevacizumab, etoposide, cisplatin, *IT MTX* intrathecal methotrexate

The median OS of all 34 patients was 5.2 months (95% CI 2.2–9.7); 31 had died when survival data were collected (Fig. 1a). Survival rates at 1 and 2 years were 29% and 10%, respectively. In univariate analyses (Table 2), intrathecal methotrexate was significantly associated with better OS. Although improved survival with systemic treatment was not statistically significant (p = 0.070), breast cancer patients with LM who received BEEP had significantly prolonged survival compared to those treated with other regimens; the median OS of patients who received BEEP regimens was 9.7 months compared with 1.4 months for those on non-BEEP regimens (p = 0.002). Another significant prognostic factor was previous stereotactic radiosurgery for brain metastases. ER or HER2 status were not significantly associated with OS. Breast cancer subtype and brain metastases were not significantly different between BEEP and non-BEEP-treated patients (Supplementary Table 1).

**Table 2:** Univariate hazard ratios for overall survival of 34 patients with leptomeningeal metastasis

	Hazard ratio (95% CI)	p-value
Year		
2011–2013	Ref.	0.005
2014–2016	0.31 (0.14, 0.71)	
Age at leptomeningeal metastasis diagnosis		
≤ 55 years	Ref.	0.166
> 55 years	1.66 (0.81, 3.42)	
Intrathecal methotrexate vs non-intrathecal methotrexate	0.27 (0.10, 0.69)	0.006
Systemic treatment	0.50 (0.24, 1.06)	0.070
BEEP vs non-BEEP	0.32 (0.15, 0.66)	0.002
Anti-HER2 therapy (intrathecal)	0.40 (0.12, 1.36)	0.143
With vs without whole brain radiotherapy	1.41 (0.53, 3.74)	0.495
With vs without prior stereotactic radiation surgery	0.34 (0.13, 0.92)	0.033
Histology		
Lobular carcinoma	Ref.	0.101
Other carcinoma	2.17 (0.86, 5.44)	
Brain parenchyma metastasis		
None	Ref.	0.051
Metachronous	0.41 (0.16, 1.03)	
Synchronous	1.26 (0.54, 2.92)	
Breast cancer subtype		
ER+/HER2−	Ref.	0.894
HER2+	0.80 (0.32, 2.01)	
Triple negative	0.91 (0.39, 2.10)	

*BEEP* bevacizumab, etoposide, and cisplatin regimen, *ER* estrogen receptor, *HER2* human epidermal growth factor receptor-2

We saw a trend towards increased use of systemic BEEP for breast cancer patients with LM during 2014–2016 compared with 2011–2013 (p = 0.08, Chi-Square test). In parallel, OS was significantly longer in 2014–2016 than in 2011–2013 (13.6 vs 3.2 months, p = 0.0036) (Fig. 1b).

For the two patients of three who received both intrathecal trastuzumab and intrathecal methotrexate, who also received BEEP, the median OS was 17.0 months (95% CI 15.4–24.8) (Fig. 1c).

### Treatment effects

In the adjusted Cox proportional hazard model (Table 3), BEEP remained a significant prognostic factor for OS. The impact of intrathecal MTX became non-significant but intrathecal trastuzumab had significant prognostic impact for HER2-positive patients. The survival curves of BEEP (all received intrathecal methotrexate), intrathecal methotrexate without BEEP, and no treatment were shown in Fig. 1c.

**Table 3:** Adjusted hazard ratios of treatments for leptomeningeal metastasis in 34 patients

	Adjusted hazard ratio (95% CI)	p-value
Intrathecal methotrexate vs non-intrathecal methotrexate	0.86 (0.28, 2.64)	0.787
BEEP vs non-BEEP	0.24 (0.09, 0.62)	0.003
Intrathecal trastuzumab vs no intrathecal trastuzumab	0.22 (0.05, 0.90)	0.035

*BEEP* bevacizumab, etoposide, and cisplatin regimen, *HER2* human epidermal growth factor recptor-2

Table 4 compares the treatment response rates and outcomes of patients with different breast cancer subtypes and who received different treatment modalities. Patients who had a CSF response had significantly better OS than those who did not (HR 0.25, 95% CI 0.11–0.55, p < 0.001).

**Table 4:** Responses of 34 patients to treatment for leptomeningeal metastasis

	CSF response		p-value^a^
	Number (%)	OR* (95% CI)	
All patients	19/34 (58%)	–	
ER+	13/21 (61.9%)	1.90 (0.47, 7.70)	0.484
HER2+	6/7 (85.7%)	6.46 (0.68, 61.16)	0.104
TNBC	3/10 (30.0%)	0.21 (0.04, 1.06)	0.068
Median time to CSF response (days)	28 [8, 138]		
CSF response to intravenous BEEP plus intrathecal methotrexate vs to monomodal or no treatment		OR = 3.25 (0.79, 13.30)	0.100
Overall survival of patients with vs without a CSF response		HR = 0.25 (0.11, 0.55)	< 0.001

*CSF* cerebrospinal fluid, *ER* estrogen receptor, *HER2* human epidermal growth factor receptor-2, *TNBC* triple-negative breast cancer, *BEEP* bevacizumab, etoposide, and cisplatin regimen, *OR* odds ratio, *HR* hazard ratio

^a^For each subtype, a 2 × 2 table of all patients were constructed, fisher exact test was performed, and OR was calculated

## Discussion

To the best of our knowledge, this is one of the largest reported cohorts to have received the systemic BEEP regimen after diagnosis of LM. We have affirmed that breast cancer with LM responds to treatment and that using systemic BEEP is associated with prolonged OS, potentially widening the efficacy of BEEP to include this indication. Furthermore, we also observed various patients who had a dramatic neurological recovery after systemic BEEP treatment. Although the details of quality-of-life was not recorded in the medical chart, the impressive changes in functional status implied that systemic treatments should still be considered and discussed for LM patients with deteriorating performance status.

Vascular endothelial growth factor has been implicated in LM progression [17], and higher levels in CSF are associated with worse prognosis of melanomas and breast cancer with LM [18]. Antiangiogenic therapy potentiates vascular normalization that may facilitate the transit of chemotherapeutic agents [19]; this effect has been shown to improve the perfusion and delivery of antiangiogenic drugs to treat glioblastoma [20]. In our previous study, more substantial changes in K^trans^, a biomarker for vascular permeation as measured by dynamic contrast enhanced magnetic resonance imaging, after 24 h of bevacizumab infusion was associated with longer control of brain parenchymal metastatic tumors in MBC [21, 22]. Although our previous findings were limited to MBC patients with brain parenchyma metastases, the fact that three out of four systemic chemotherapies without concomitant bevacizumab were not associated with an improved outcome in LM provides further indirect evidence that the efficacy of the BEEP regimen in LM may also related to vascular normalization.

Despite the potential risk of leukoencephalopathy in patients treated with intrathecal methotrexate, and we did not identify any risk factors associated leukoencephalopathy; our study also showed that intrathecal methotrexate may not be as important as systemic chemotherapy for treating LM. Although intrathecal methotrexate may alleviate neurological symptoms caused by a large tumor cell load, other studies suggest that intrathecal cytotoxic treatment may not be as efficacious as previously supposed [6, 8, 9, 11]. A systemic review concluded that intrathecal cytotoxic agents have no real benefit and increase the risk of adverse effects [6]. However, patients in most retrospective studies, including ours, received intrathecal cytotoxic agents and systemic treatment concurrently, so we cannot rule out a synergistic effect of intrathecal and systemic treatment. Prospective studies with BEEP alone or with intrathecal methotrexate strategies are necessary to clarify the role of intrathecal cytotoxic agents in treating LM.

Conversely, intrathecal trastuzumab was associated with significantly improved survival in three of our seven HER2-positive patients; notably, none of them received intravenous trastuzumab, supporting attribution of the benefit of trastuzumab to intrathecal delivery. Several case reports have found intrathecal trastuzumab efficacious for patients with HER2-positive breast cancer with LM refractory to intravenous trastuzumab-based regimens [10]. A recent phase I study of intrathecal trastuzumab for LM confirmed its safety, with a recommended phase II dose of 150 mg/week [23]; phase II clinical trials are ongoing and we eagerly await the results.

A higher proportion of patients in our study than in the general primary breast cancer population had lobular carcinoma (23.5% vs 10.0%), similar as reported in other LM studies of breast cancer [3]. Interestingly, one-third developed the first metastatic site in the leptomeninges without brain parenchymal metastases; despite LM, another third had synchronous brain metastasis and 29.4% had metachronous brain metastasis prior to LM diagnosis. Furthermore, different patterns of CNS involvement associated with LM had different prognosis, suggesting that the underlying pathogenesis of metastatic breast cancer and LM may not entirely overlap; for example, patients with LM not involving the parenchyma had worse survival than those with brain metastases followed by metachronous LM. Notably, patients who had stereotactic radiosurgery for brain metastasis had survived better after LM was diagnosed. Although the use of stereotactic radiosurgery may be governed by the numbers of metastatic tumors, other possible immunogenic or abscopal effects of stereotactic radiosurgery may also explain improved survival with prior treatment [24]. Boire et al. recently demonstrated that activation of the immune compliment system is associated with LM but not brain metastasis, in humans and mouse models [25], suggesting that we may need to rethink the treatment of LM patients.

Our study had limitations, foremost the caveat of selection bias inherent in retrospective studies. However, we endeavored to minimize this possibility by objectively including all patients with LM in the NTUH cytopathology database. As it is our policy to do CSF testing for every LM case suspected for clinical or radiological reasons, every cytological CSF assessment would leave a record. Efficacy of BEEP regimen against LM in only 34 patients may also be questioned; however, objective endpoints such as OS and response by consecutive CSF cytology results substantiate our results. Moreover, a multivariate model to differentiate the contribution of each treatment modality also affirmed superior activity of the BEEP regimen. On the other hand, all patients who received the BEEP regimen also received intrathecal methotrexate, so research is warranted to investigate whether the effect of BEEP is synergistic with intrathecal treatment. Secondly, although intrathecal trastuzumab was associated with better OS, none of the patients with HER2-positive breast cancer with LM received intravenous trastuzumab, and continued systemic anti-HER2 treatment after CNS metastases may be associated with improved survival [2]. However, as Taiwan National Health Insurance does not reimburse trastuzumab after progression on first-line treatment, trastuzumab would be expensive to buy out-of-pocket. Our results suggest that intrathecal trastuzumab may an alternative for patients with HER2-positive breast cancer with LM who cannot afford intravenous trastuzumab. Lastly, the results of OS may be subject to lead-time bias if the criteria to initiate diagnostic or treatment procedures are different from patient-to-patient. However, all the patients in this study were treated by the same breast medical oncologists through the time periods as such that the criteria for LM diagnosis and treatment would be similar across the patients, limiting the chance of lead-time bias.

## Conclusions

Using a non-biased study design to evaluate systemic therapy for breast cancer with LM in a real-world clinical setting, we have shown that LM is a treatable, with a median OS of 9.63 months for patients receiving systemic BEEP treatment. Intrathecal methotrexate may not have additional benefit with the BEEP regimen. Nonetheless, intrathecal trastuzumab is associated with significantly improved OS in patients with HER2-positive breast cancer with LM. A clearer understanding of the role of systemic regimens, especially BEEP, in LM is needed to improve the management and prognosis of breast cancer with LM.

## Electronic supplementary material

Below is the link to the electronic supplementary material.

Supplementary file1 (DOCX 16 kb)

## Data Availability

The datasets generated and analyzed during the current study are not publicly available due no permission from the authorized institution, but are available from the corresponding author on reasonable request.
