# Supplementary material for: Systemic treatment of breast cancer with leptomeningeal metastases using bevacizumab, etoposide and cisplatin (BEEP regimen) significantly improves overall survival
Source: J Neurooncol. 2020 Apr 29;148(1):165–72. doi: 10.1007/s11060-020-03510-y (PMC7280357; doi:10.1007/s11060-020-03510-y)
Supplement: Supplementary file 1 — Supplementary file1 (DOCX 16 kb) [file 11060_2020_3510_MOESM1_ESM.docx]

Supplementary Table 1 The breast cancer subtype and brain parenchymal metastasis between first-line BEEP-treated and non-BEEP treatment LM patients

|  | BEEP  (n = 19) | Non-BEEP  (n = 15) | P value* |
| --- | --- | --- | --- |
| Breast cancer subtype |  |  | 0.07 |
| TNBC | 8 | 2 |  |
| Non-TNBC | 11 | 13 |  |
| Brain parenchymal metastases |  |  | 0.33 |
| Synchronous brain parenchyma metastasis | 8 | 6 |  |
| Metachronous brain parenchyma metastasis | 6 | 2 |  |
| No brain mesenchyma metastasis | 5 | 7 |  |

* P values were calculated by Chi-squared test

BEEP: bevacizumab, etoposide, and cisplaitn regimen, LM: leptomeningeal, TNBC: triple negative breast cancer;
